# Effect of acute interval sprinting exercise on postprandial lipemia of sedentary young men

**DOI:** 10.20463/jenb.2016.03.20.1.7

**Published:** 2016-03-31

**Authors:** Aaron Chu, Yati N Boutcher, Stephen H Boutcher

**Affiliations:** 1 School of Medical Sciences, University of New South Wales, SydneyAustralia

**Keywords:** Postprandial Lipemia, High-fat Meal, Interval Exercise, Sprint Cycling

## Abstract

**[Purpose]:**

Postprandial lipemia (PPL) contributesto the development of atherosclerosis. In females, repeated 8-second bouts of interval sprinting exercise reduced PPL, however, the effect of 8-second bouts of interval sprinting on PPL of overweight males is undetermined. Thus, the effect of 8-secondsof interval sprinting for 20 min, the night before ingestion of a high-fat meal (HFM), on plasma triacylglycerol(TG) levelswas examined.

**[Methods]:**

Ten overweight males acted as participants (BMI = 26±3.0kg/m2, age 22 ± 2.5 years). A crossover design was employed withinterval sprinting and a noexercise condition separated by 7days. Participants consumed a milkshake (high-fat meal;HFM = 4170 kJ/993 Kcal) the morning after an overnight fast, followed by 4 hourly blood samples. Participants performedone bout of interval sprinting (8seconds sprinting at 110-115rpm, 12seconds active recovery at ~60rpm for 20 minutes) the evening before the consumption of the HFM.

**[Results]:**

Postprandial TG was 22.5% lower in the interval sprinting compared to the noexercise condition when comparing the change in total area under the curve (ΔAUCT): ISE(7.15±1.90mmolL^-1^h^-1^) versus noexercise (9.22±3.44mmolL^-1^h^-1^), p=.014. The correlation between fasting TG levels in the noexercise condition and total reduction in AUCT between the conditions was significant (r=.87, p=.001).

**[Conclusion]:**

One 20-min bout of interval sprinting,the night before consumption of a HFM,significantly attenuated the PPL response of sedentary males.

## INTRODUCTION

Fasting plasma triacylglycerol (TG) has been used as a major marker of clinical risk for the development of atherosclerosis^1^. The notion of atherosclerosis as a postprandial phenomenon, however, was first proposed by Zilversmit in the late seventies^2^. Postprandial lipemia (PPL) refers to the temporary elevation of plasma triacylglycerol after the consumption of a fat-containing meal^3^ and has recently been recognized as a more significant clinical risk factor for atherosclerosis compared to fasting TG^4^. In developed countries the widespread consumption of snacks and meals containing high levels of saturated fat results in many people spending a considerable portion of their day in the postprandial state^5^ consequently, it is important to identify lifestyle interventions that are able to lower PPL.

One such intervention is a bout of aerobic exercise that has been shown to lower TG levels in the postprandial state^6,7^ Aerobic exercise has typically involved exercising continuously on a stationary cycle ergometer at a moderately hard intensity for at least 40 min^8^. The major mechanisms underlying the aerobic exercise TG lowering effect has been suggested to be an increased removal of circulating TGs by skeletal muscle lipoprotein lipase (LPL) and a reduction in liver produced TGs entering the circulation^9^. Decreased hepatic very low density lipoprotein production (VLDL) may also contribute to the lowering TG effect as exercise increases hepatic fatty acid fat oxidation resulting in decreased secretion of VLDL^10^.

Although aerobic exercise lasting at least 40 min reduces PPL performing multiple sessions per week is likely to be challenging for the majority of individuals as the most frequent reported reason for not exercising is ‘lack of time’^11^. Recently, however, there has been increased interest in a time-efficient exercise modality known as interval sprinting exercise (ISE). ISE involves repeated bouts of all-out sprinting alternating with low-intensity exercise or rest. The duration of the sprint and recovery phases of ISE protocols has ranged from 6 s to 30 s. ISE protocols such as the 30 s Wingate test (ISE-30) are extremely demanding and for untrained, overweight individuals are difficult to complete^12^. Therefore, long term studies investigating fat loss using the ISE protocol have not been carried out. Twelve to 15 weeks of moderate intensity interval sprinting exercise (ISE-8), involving repeated 8 s sprints with 12 s recovery periods, however, has been shown to result in a significant reduction of total body fat^13,14^ and visceral fat^15^. The ISE-8 protocol involves an 8-s sprint followed by 12 s of easy pedaling, repeated for 20min, in contrast to the Wingate test (ISE-30) which typically consists of 4 maximal exertion 30 s sprints with 3 to 4 minutes recovery between sprints.

The ISE-30, repeated 4-5 times, with 3-4 min rest intervals, has shown to be effective in reducing plasma TG after the consumption of a standardized fatty meal^16^. Using the ISE-8 protocol Tan et al.^18^ also found that 20 min of ISE-8 resulted in a 13% lowering in blood TG levels after consumption of a high fat meal (HFM) 14 h post exercise. Participants in this study were young females possessing normal fasting TG levels. The 13% decrease in PPL is lower than that found in ISE-30 studies which have produced a decrease of about 21%^7^. Whether the ISE-8 protocol, compared to the ISE-30 protocol, also results in a lower PPL effect in sedentary males, however, is undetermined. Therefore, the aim of the study was to examine the effect of 20 min of ISE-8 on the PPL response of untrained males. It was hypothesized that a single bout of ISE-8 would lead to significant PPL attenuation.

## METHODS

### Participants

Ten untrained males acted as participants for the study which was approved by a University Human Research Ethics committee. Participants were classified as untrained if they reported exercising for less than 3 sessions per week at a light intensity. Participants were excluded if they exercised regularly or had a significant history of cardiovascular and respiratory disease, smoking, chronic metabolic disease, or lactose or fat intolerance. Possible contraindications to exercise were screened using the Physical Activity Readiness questionnaire (PAR-Q).

### Maximal oxygen uptake test

A $\dot{V}$O_2_max test was performed on a Monark Ergomedic 839E ergometer (Monark, Vansbro, Sweden) with a closed respiratory gas exchange system usinga True Max 2400 Metabolic Cart (ParvoMedics Inc, USA). The test began with a 3-min warm-up at an initial load of 30 watts (W) and 60 revolutions per min (rpm).After the first 3 min, the power output was increased at a rate of 30W/min.The test was completed when the following criteria were fulfilled:( 1) inability to retain a pedaling cadence of 60pm, (2) plateauing in $\dot{V}$O2response despite increasing exertion, (3) respiratory exchange ratio (RER) ≥ 1.15, and (4) heart rate (HR) within 10 bpm of age-predicted maximum (220 - age)^19^. Min-by-minmeasurements of HRand Borg’s rate of perceived exertion (RPE) were also recorded^20^. HR was monitoredusing short range telemetry (Polar S810I, Polar Electro, Kempele, Finland).

### Experimental design

A crossover counterbalanced study design was employed to compare the PPL response between the no exercise (No Ex) and exercise (Ex) conditions that were separated by a minimum of 7 days. In the Ex condition, each participant performed a 20-min ISE-8 session (30 min with 5 min of warm-up and cool-down) the evening before the fat feeding session which occurred on the following morning. Participants were asked to keep a 3-day food diary (2 week days and 1 weekend day) (SERVE Nutrition Management System, version 5.1.002, 2004, Australia) and were instructed to avoid physical exercise, alcohol, and caffeine consumption 3 days before each testing session.

### Blood sampling and analysis

A 12-h overnight fast was required before the initiation of both conditions. Participants reported to the laboratory between 7 am to 9 am the following day and completed a questionnaire to check adherence to dietary and lifestyle restrictions. A 22-gauge cannula (Becton Dickinson, Plymouth, UK) was inserted into an antecubital vein to allow continuous whole blood sampling. A 3-way stopcock (Becton Dickinson, Plymouth, UK) was used to enable the attachment of a 10 mL EDTA vacutainer for blood sample collection and a syringe filled with 0.9% isotonic saline (Pfizer, New York, USA) for flushing to maintain cannula patency.

Baseline fasting blood samples were analyzed using enzymatic photometric assay (AccutrendBlood® Plus System, Roche, Germany) to establish TG levels. Subsequent samples were collected and analyzed at each hour after the administration of the HFM. Participants were asked to ingest the HFM within 15 min. Also whole blood samples were obtained at baseline during the No Ex condition via a 22-gauge cannula (Becton Dickinson, Plymouth, UK) into a 10 mL EDTA vacutainer (Becton Dickinson, Plymouth, UK) and cholesterol levels were assessed by automatic enzymatic reactions using a Cholestech analyzer (Cholestech LDX, Hayward, California, USA).

### High-fat meal

The HFM was in milkshake form and consisted of 275 g of thickened cream (Coles, Australia), 50 g of specialty ice cream (Sanitarium, Australia), and 2 g of sweetener (Equal, Merisant, USA). This amounted to 4170 kJ of energy, 98 g of fat (63.6 g saturated), 24 g of carbohydrate, and 8.4 g of protein.

### Exercise session

**Figure 1.**
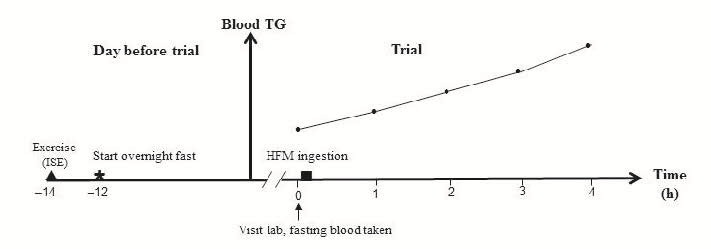
Diagrammatic representation of the study design. In the exercise condition, participants were required to perform one ISE-8 session 14 h prior to blood sampling the next morning.

The ISE-8 protocol was performed by all participants during the evening, 14.8 h (± 1.17) before ingesting the HFM the next morning. The ISE-8 session involved participants pedaling on a Monark cycle ergometer (Ergomedic 839E, Sweden) for 20 min, along with 5 min of warmup and cool-down. A pre-recorded ISE-8 sound track was played to assist the participants to keep the correct cadence throughout the session. During the warm-up phase, the participants pedaled at 60 rpm against 0.5 kg resistance. Following the warm-up phase, all participants pedaled against a pre-determined cycling load at 60% of their maximum power output during the 8 s sprinting phase and approximately 60 rpm during the 12 s active recovery phase, repeatedly for 20 min. Mean power was calculated from the product of the cadence and the cycling resistance during the ISE-8 session. Figure 1 illustrates the time line of the trial.

### Statistics

The totalarea under the curve (AUCT) for both NoEx and Ex conditions was calculated using the trapezoidal rule(SPSS Inc, Illinois, USA). The incremental area under the curve (AUCI) for both conditions was obtained by subtracting the area under baseline TG from the TG at each hour for each condition. A 2 by 5 (condition versus hrs) two-way ANOVA was performed to analyze the interactions between time and condition on the level of TGs.Paired student t-tests were used to analyze the time effect on the levels of TG for both conditions. Huynh-Feldt adjustments were utilized if Mauchly’s test of sphericity was violated. Results were considered significant if probability was less than .05. Effect size was determined using Eta squared (η^2^). Values of 0.1, 0.3, and 0.5 were used for small, medium, and large effect sizes. Experimental and descriptive data are presented as mean ± standard deviation of the mean (SD).

## RESULTS

### Participants

Anthropometric characteristics of the participants are summarized in Table 1. Lipid profiles for the participants are reported in Table 2.

The mean power output during the 8-s sprint was 152 W (Table 3) and during the 12-s recovery was 78 W. The average RPE during the 20-min of ISE-8 was 14 ± 1.6 and the average HR was 163 ± 15.3 bpm.

**Table 1.** Participant characteristics

Characteristics	Mean (± SD)
Age (years)	22 ± 2.40
Body mass (kg)	79.9 ± 13.4
Height (m)	1.75 ± 0.09
Body-mass index, BMI (kg/m2)	26 ± 2.97
Waist circumference (cm)	92 ± 7.36
Hip circumference (cm)	100 ± 8.12
Waist-hip ratio	0.92 ± 0.03
Body fat percentage (%)	19 ± 5.25
$\dot{V}$O_2_O_2_max (ml/kg/min)	40.4 ± 8.63
Systolic blood pressure (mmHg)	130 ± 6.98
Diastolic blood pressure (mmHg)	76 ± 8.47

**Table 2.** Participant fasting measurements

Characteristics	Mean (± SD)
Total cholesterol (mmol/L)	4.49 ± 0.66
TG (mmol/L)	1.64 ± 0.38
LDL (mmol/L)	3.03 ± 0.60
HDL (mmol/L)	0.99 ± 0.22
TC/HDL Ratio	4.37 ± 0.82
Glucose(mmol/L)	4.94 ± 0.35

**Table 3.** Cycling data during the 20 minutes of interval sprinting exercise

Cycling Data	Mean (± SD)
Mean power (W) during the 8 s sprinting phase	152 ± 29.5
Mean cadence (rpm) during the 8 s sprinting phase	117 ± 2.40
Mean pedal resistance (kg) during the 8 s sprinting phase	1.3 ± 0.25
Mean heart rate (bpm) throughout exercise	163 ± 15.26
Mean rating of perceived exertion during exercise	14 ± 1.45

### Dietary analysis

During the 3 days before each condition participants’ average total energy consumption was 11720 (± 2961) kJ with 5640 (± 3192) kJ of carbohydrate, 2670 (± 1134) kJ of protein, and 5090 (± 2756) kJ of fat.

### Postprandial triacylglycerol response

**Figure 2.**
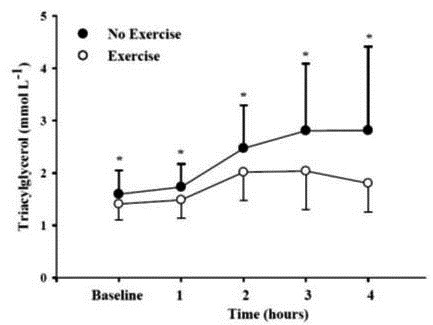
The postprandial response for the No Ex and Ex conditions. Bars represent SD. *Significant time effect, condition effect, and time and condition interaction.

**Figure 3.**
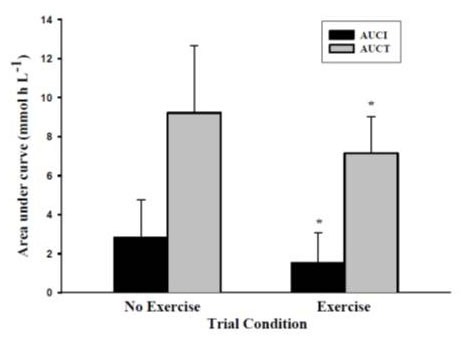
The area under the curve incremental (AUCI) and area under the curve total (AUCT) values for the No Ex and Ex conditions. Bars represent SD. *Significant difference was found for ΔAUCT (22.5%) and ΔAUCI (46.4%).

Following the HFM, a significant increase in plasma TG was observed in both conditions, p = .001 (Figure 2). A significant condition effect was also identified, p = .02, with the Ex condition producing lower TG levels. Difference between the mean of AUCT-Ex and AUCT-No Ex was significant (p = .014) with a large effect size (η^2^ = 0.51) (Figure 3). The mean of AUCT-Ex, 7.15 ± 1.86 mmol L^-1^ h^-1^, was significantly lower (22.5%) than the mean of AUCT-No Ex, 9.22 ± 3.44 mmol L^-1^ h^-1^. The difference between the mean of AUCI-Ex and AUCI-No Ex was also significant (p = 0.04) with a medium effect size (η^2^ = 0.37). The mean of AUCI-Ex, 1.52 ± 1.55 mmol L^-1^ h^-1^, was also significantly lower (46.4%) than the mean of AUCT-No Ex, 2.83 ±1.93mmol L^-1^h^-1^.A paired sample t test was used to examine the fall of TG concentration at the 3rd and 4th hour of the exercise condition. Asignificant fall in TG concentration was observed (11.5%, p=.038,η^2^ = 0.40).A significant positive correlation was also found between fasting TG in the NoEx condition and total reduction in AUCT between the conditions(r=.87, p=.001).A significant positive correlation was also found between fasting TG and AUCT in the No Ex condition (r= .92, p= .0001).

## DISCUSSION

An acute bout of ISE-8, the evening before a HFM, significantly reduced PPL in comparison to a No Ex condition. The difference in AUCT-TG between the two conditions was 22.5% which is consistent with past ISE-30 studies. A significant correlation also existed between fasting TG levels in the No Ex condition and total reduction in AUCT between the conditions. This study is the first to show that one bout of ISE-8 was able to significantly reduce fasting TG and total PPL response of untrained males.

The 22.5% AUCT reduction in this study was similar to two previous studies that showed a 21% and 18% reduction of TG AUCT after the ISE-30 protocol^21,17^. Collectively, these ISE-8 results extend prior research that has documented a consistent reduction in PPL after moderate-intensity aerobic exercise6,7. A smaller PPL effect (13%) was found by Tan et al.^18^ who administered the same ISE-8 protocol as used in the present study with 12 young physically inactive females. It has been shown that the PPL of inactive premenopausal females is significantly smaller than that recorded by inactive males^22,23,24^. The lower PPL of women compared to men could be brought about by increased clearance of circulating TG, a slowed absorption of dietary fat, or a decreased production of VLDL-TG in the postprandial state^25^. Horton et al.^25^ have shown that the enhanced skeletal muscle clearance of lipoprotein TG of women contributes to their lower PPL. As it has been also shown that skeletal muscle LPL activity is similar in men and women^26^ it appears other mechanisms may regulate the lipemic response to fat ingestion in men and women. Horton et al.^25^ have pointed out that the PPL response of postmenopausal women is exaggerated compared to premenopausal women and thus have speculated that endogenous estrogen production may contribute to gender differences in PPL.

Another possible explanation for gender differences in PPL could be the clearance of circulating VLDL and chylomicrons via a saturable pathway^27^. It has been shown that the higher fasting TG found in males compared to females was significantly correlated to their greater postprandial excursion^27,28^. Males in the present study also demonstrated this relationship as fasting TG was significantly correlated (r = .92) with their PPL response in the No Ex condition. Interestingly, males in the present study also demonstrated a strong relationship between fasting TG in the No Ex condition and total reduction in AUCT between the conditions (r = .87). Thus, males with greater resting TG levels demonstrated a greater PPL reduction response after ISE-8 compared to the No Ex condition. Although the mechanism underlying the greater PPL response of males is undetermined both fasting TG and PPL response have been related to visceral adiposity in men22. Thus, males possessing elevated visceral adiposity tend to have higher fasting TG levels and a greater PPL response, however, males still exhibit exaggerated PPL when their PPL response is compared with females possessing similar fasting TG levels23. Therefore, further studies are required to explain potential differences in the regulation of the PPL response of males and females.

There was a notable reduction in TG levels at the third and fourth hours in the Ex compared to the No Ex condition (Figure 2). The ΔAUCI was also significantlygreater (46.4%) in the Ex compared to the No Ex condition. Freese et al.^21^ examined the effect of prior exercise on PPL and also found a significant AUCIreduction in TG (23%) after anISE-30exercise protocol. The reduction of TG at the 4th hourin anaerobic exercise condition, but not in a noexercise condition, has also been observed by others who investigated the effects of prior aerobicexercise on PPL^29,30,31^.

Several mechanisms have been proposed to underlie the reduction of AUCT-TG after a single bout of ISE. For example, ISE could reduce PPL by increasing TG clearance via the LPL pathway. LPL activity has been suggested to be the prime mechanism for the reduction in plasma TG during the postprandial state^7,9^. Also high-intensity aerobic interval training has been shown to enhance the reduction effect of plasma VLDL-TG for up to 48 h^32^. Furthermore, Gabriel et al.^17^ proposed that LPL-expression could be muscle-fiber type dependent. High numbers of fast fiber recruitment in ISE could lead to greater LPL expression and generate a greater TG clearance, however, this hypothesis has not yet been examined in humans.

Bellou et al.^33^ examined the effect of high-intensity aerobic interval exercise on the hepatic pathway of TG metabolism 14 h after exercise, and found a significant 21% reduction in VLDL-TG concentration. This was interpreted as an increase in plasma VLDL-TG clearance rate as the level of hepatic VLDL secretion remained unchanged. Thus, ISE could also lead to the production of concentrated VLDL particles which have a higher affinity for LPL^34^. Whatever the mechanism the ability of ISE to significantly reduce PPL would seem to have positive implications for the prevention of atherosclerosis^7^.

### Limitations

Several limitations exist for the current study. As the participants tested in this study were healthy young adults, it is uncertain if these results will be directly translatable to less healthy populations with multiple comorbidities. Also, only the effect of a single bout of ISE-8 on PPL response was examined. Thus, it is unclear if chronic bouts of ISE-8 will have a similar PPL attenuation effect.

## CONCLUSION

In conclusion, the ISE-8 protocol was shown to be effective in attenuating the PPL response in sedentary males when compared to a no exercise control condition. The PPL attenuation effect of ISE-8 observed in the current study is consistent with other ISE-30 experiments conducted. When the results are compared to a previous study performed at the same laboratory with sedentary females, however, a greater PPL attenuation was observed.

## AUTHOR CONTRIBUTIONS

Conceived of and designed the experiment: Steve Boutcher and Yati Boutcher. Performed the experiment: Aaron Chu. Analyzed the data: Aaron Chu and Yati Boutcher. Wrote the paper: Steve Boutcher, Aaron Chu, and Yati Boutcher.
